# Arf GAPs as Regulators of the Actin Cytoskeleton—An Update

**DOI:** 10.3390/ijms20020442

**Published:** 2019-01-21

**Authors:** Christine E. Tanna, Louisa B. Goss, Calvin G. Ludwig, Pei-Wen Chen

**Affiliations:** Department of Biology, Williams College, Williamstown, MA 01267, USA; cet4@williams.edu (C.E.T.); lbg2@williams.edu (L.B.G.); calvinludwig95@gmail.com (C.G.L.)

**Keywords:** actin, ADP-ribosylation factors, ADP-ribosylation factor GTPase-activating protein, focal adhesion, podosome, phagocytosis, ASAP1, ARAP1, ARAP2, GIT1/2

## Abstract

Arf GTPase-activating proteins (Arf GAPs) control the activity of ADP-ribosylation factors (Arfs) by inducing GTP hydrolysis and participate in a diverse array of cellular functions both through mechanisms that are dependent on and independent of their Arf GAP activity. A number of these functions hinge on the remodeling of actin filaments. Accordingly, some of the effects exerted by Arf GAPs involve proteins known to engage in regulation of the actin dynamics and architecture, such as Rho family proteins and nonmuscle myosin 2. Circular dorsal ruffles (CDRs), podosomes, invadopodia, lamellipodia, stress fibers and focal adhesions are among the actin-based structures regulated by Arf GAPs. Arf GAPs are thus important actors in broad functions like adhesion and motility, as well as the specialized functions of bone resorption, neurite outgrowth, and pathogen internalization by immune cells. Arf GAPs, with their multiple protein-protein interactions, membrane-binding domains and sites for post-translational modification, are good candidates for linking the changes in actin to the membrane. The findings discussed depict a family of proteins with a critical role in regulating actin dynamics to enable proper cell function.

## 1. Introduction

Coordinated changes in the membrane and actin cytoskeleton underlying several motile cellular structures are critical for normal physiology, including development, wound healing and phagocytosis and contribute to pathological behaviors such as cancer invasion and metastasis [1,2,3]. The polymerization of actin filaments against membranes provides a force to drive membrane protrusions such as lamellipodia of a migrating cell or phagocytic cups in macrophages engulfing bacteria. In addition to actin dynamics, the architecture of the actin structures is also carefully controlled. For example, actin filaments cross-linked by nonmuscle myosin 2 (NM2) form the contractile structures to retract the trailing end of cells or to divide cells. Conversely, changes in the membrane composition (i.e., phosphoinositides, membrane-associated proteins) or curvature also affect the actin dynamics and organization [4,5]. The molecular bases for spatial and temporal control of cellular membrane and actin remodeling in response to a plethora of signals are still being discovered. Though best known for their functions in vesicular trafficking, the ADP-ribosylation factors (Arfs) are increasingly recognized as important regulators of the actin cytoskeleton [6,7,8]. In this review, we discuss the literature supporting the emerging role of the Arf GTPase-activating proteins (Arf GAPs) as part of the interface between the signaling cascade and cellular machinery for remodeling of the membranes and the actin cytoskeleton.

## 2. Actin-Based Structures and Functions Affected by Arf GAPs

Arf GAPs associate with and/or affect several actin cytoskeletal structures, including focal adhesions (FAs), podosomes, invadopodia, lamellipodia, circular dorsal ruffles (CDRs) and phagocytic cups [9,10,11,12] (Table 1). FAs, podosomes and invadopodia form on the ventral surface of cells and are points of contact that link the intracellular actin network to the extracellular matrix (ECM). FAs are the larger and mature form of the integrin adhesion complexes that contain activated integrins and a large cytoplasmic plaque composed of proteins that mediate the engagement of the actin filaments with integrins as well as initiate signaling that is important for proliferation, survival and differentiation [13,14,15,16]. Some of the key FA proteins include focal adhesion kinase (FAK), paxillin, talin and vinculin. FAs are often at the ends of the actin stress fibers, which are contractile bundles of actin filaments cross-linked by NM2. Podosomes and invadopodia are highly related structures with dense cores of actin filaments (F-actin) that appear as dots, circular rosettes or belts depending on cell type [17,18]. Podosomes are found in monocyte-derived cells such as macrophages and osteoclasts, and in fibroblasts transformed by the oncogenic protein Src, while invadopodia are found in invasive cancer cells. In osteoclasts, each podosome first assembles into a circular array called a “podosome rosette”. These rosettes subsequently coalesce with each other to form a “podosome belt” that establishes a sealing zone, separating the ventral membranes from those facing the bone digesting area. Besides the attachment of the cells to the substrates, podosomes and invadopodia also mediate the degradation of the ECM and bone by secreting proteases. 

Lamellipodia, CDRs and phagocytic cups are membrane protrusions driven by actin polymerization [15,19,20,21,22]. Lamellipodia are thin membrane extensions at the leading edge of migrating cells, which contain branched actin networks associated with short-lived FA precursors called nascent adhesions. CDRs are ring-shaped, F-actin-rich structures that form on the dorsal surface of cells in response to stimulation by growth factors such as the platelet-derived growth factor (PDGF), epidermal growth factor (EGF) and hepatocyte growth factor (HGF). Two main functions have been proposed for CDR-mediated internalization: the downregulation of growth factor receptor signaling by internalization and sequestration of the ligand-bound receptors; and the transition of cells from a static to a motile state by internalizing and then recycling integrins to the ventral surface of the cells to facilitate adhesion turnover. Both functions involve the bulk internalization of cell surface receptors via macropinocytosis [23,24]. Similar to macropinocytosis, phagocytosis is another form of endocytosis dependent on F-actin [19,22]. However, phagocytosis involves the recognition and binding of specific particles by receptors on the cell surface, which then signal to reorganize the actin cytoskeleton to form the phagocytic cups. These cylindrical membrane extensions surround and then fully enclose particles such as microbes or apoptotic cells, fated for ingestion by professional phagocytic cells, mainly macrophages and neutrophils but also fibroblasts or epithelial cells. Therefore, phagocytosis plays a critical role in innate and adaptive immunity and tissue remodeling.

## 3. Arf and Rho Family GTP-Binding Proteins in Actin Remodeling

The ADP-ribosylation factors (Arfs) and Rho family proteins are small GTP-binding proteins in the Ras superfamily, both of which are important regulators in the signaling pathways governing complex cell behaviors such as cell migration, cell division and phagocytosis. Common to these cellular functions is the coordinate change in membranes and the cytoskeleton. Like other GTPases, Arf and Rho proteins switch between active GTP and inactive GDP-bound forms and interact with their effectors in the GTP-bound state. The cycling between the two forms is regulated by, among others, the guanine nucleotide exchange factors (GEFs) that promote the exchange of GDP for GTP, and GTPase-activating proteins (GAPs) that stimulate GTP hydrolysis. In this section, we describe the current understanding of the roles of Arfs and Rho proteins in regulation of the actin cytoskeleton.

In humans, 20 genes encode for Rho family proteins, with RhoA, Rac1 and Cdc42 being the best-characterized members [67,68]. Activation of each induces different actin structures [6,68]. Typically, the activation of RhoA stimulates the formation of stress fibers and FAs through the action of several effectors including mDia, ROCK (Rho-associated kinase) and NM2. Activated Rac1 forms membrane ruffles like CDRs, lamellipodia and nascent adhesions. Some of the key targets of Rac1 are PAK (p21-activated kinase) and WAVE (WASP family veroprolin homologous protein) regulatory complex (WRC). Activated Cdc42 stimulates the formation filopodia, which is mediated by PAK and N-WASP (neural Wiskott-Aldrich syndrome protein). Activated WRC and N-WASP enhance Arp2/3 complex-dependent actin polymerization that produces branched actin networks.

The Arf family includes the Arfs, Arf-like proteins (Arls) and Secretion-associated and Ras-related proteins (SARs) [69,70]. The six mammalian Arfs can be classified into three groups: Arf1, 2 and 3 (class I), Arf4 and 5 (class II) and Arf6 (class III). Arf1 and Arf6 are the most extensively studied. Both have been shown to affect the actin cytoskeleton and associated structures [8,71]. At Golgi, activated Arf1 regulates the Arp2/3-dependent actin dynamics by recruiting ARHGAP10 (later renamed as ARFGAP21), a Cdc42 GAP to control Cdc42 [72]. Arf1 is also increasingly recognized to function at the plasma membrane. Activated Arf1 cooperates with Rac1 to stimulate WAVE regulatory complex (WRC)-dependent actin polymerization [73], central for membrane extensions for *Salmonella* invasion of the host cells [74]. Similarly, the *Drosophila* Arf1 is essential for WRC-driven lamellipodia formation [75]. Activated Arf1 induce actin waves like activated Arf6 [76]. Arf1 also affects FAs and cell migration/invasion [77,78]. Numerous lines of evidence support a prominent role of Arf6 in cortical actin remodeling. Studies mostly show activated Arf6 stimulates protrusive membrane structures [8,79], including lamellipodia, macropinocytic ruffles or phagocytic cups, which typically requires the activation of Rac1. In agreement with these findings, Arf6 has been shown to function in cell migration, phagocytosis, and the disassembly of FAs [8,9,45,71,80,81].

The mechanisms by which Arfs regulate actin remodeling are still being discovered. Some effects on actin may involve membrane trafficking [7]. Arf6 is involved in the transport of Rac1 and lipid raft components necessary for Rac1 activation to the plasma membrane [82,83,84]. Arf6 can also modulate actin by recruiting Rac1 GEFs such as DOCK180/ELMO and Kalirin [85,86] or actin regulators [73,87,88]. Arf6 activates phosphatidylinositol 4-phosphate 5 kinase (PI4P5K), generating PI(4,5)P2, thereby affecting actin regulators that operate under the control of PI(4,5)P2 [1,3]. The interactions with Arf GAPs are another way that Arfs may affect actin.

## 4. The Arf GAP Family

Arf functions depend on cycling between the GTP and GDP-bound forms. Because Arfs have a low intrinsic rate of exchanging GDP for GTP and no detectable GTPase activity to hydrolyze GTP to GDP and free phosphate, cycling between the two forms must rely on Arf GEFs and Arf GAPs. However, studies examining the role of the Arf GAPs in various cellular processes, including ones discussed in this review, argue for roles of the GAPs acting beyond inactivating Arfs, by functioning as Arf effectors or as Arf cyclers via working with specific Arf GEFs to promote Arf functions.

**Figure 1:**
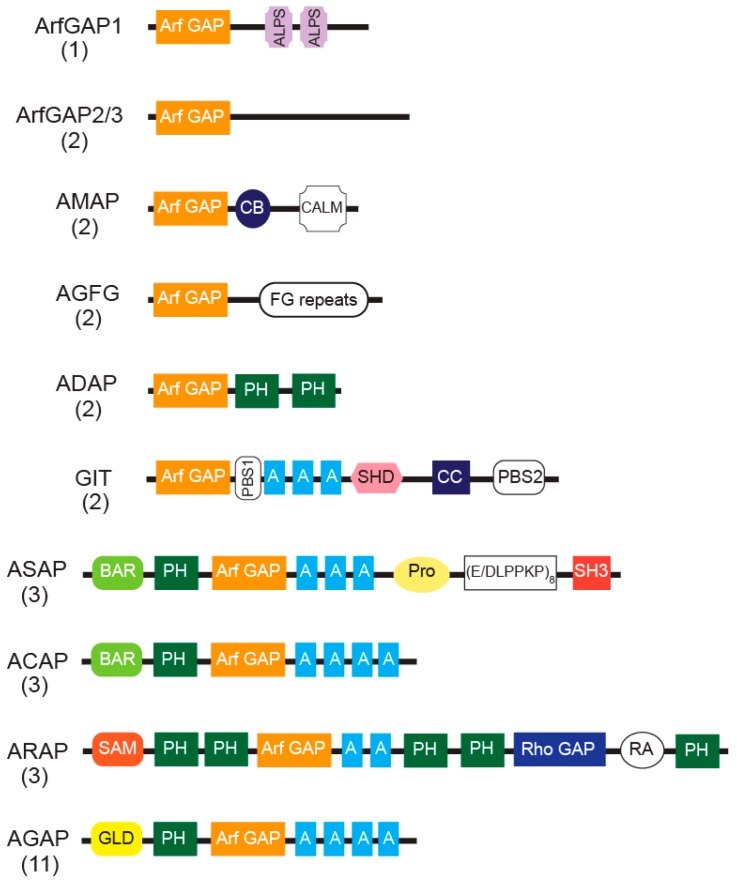
The domain structures of Arf GAPs. Representative domain organizations of each human Arf GAP group are depicted but not to scale. The number in parentheses below the name of the subfamily indicates the number of members in each Arf GAP subfamily. Arf GAP, Arf GTPase-activating domain; ALPS, ArfGAP1 lipid-packing sensor; CB, clathrin-box; CALM, CALM binding domain; FG repeats, multiple copies of the XXFG motif; PH, pleckstrin homology domain; A, ankyrin repeat; PBS, Paxillin binding site; SHD, Spa-homology domain; CC, coiled-coil; BAR, Bin/Amphiphysin/Rvs; cluster of three Proline-rich (PxxP) motifs; (E/DLPPKP)8, eight tandem (E/DLPPKP) motifs; SH3, Src homology 3 domain; SAM, sterile α-motif; RhoGAP, RhoGAP domain; RA, Ras association motif; GLD, GTP-binding protein-like domain. SMAP2 has CALM, but SMAP1 does not. ASAP1 contains the Pro (E/DLPPKP) repeat but ASAP2 and ASAP3 do not. ASAP3 lacks an SH3 domain.

Arf GAPs are defined by the catalytic Arf GAP domain, which stimulates the hydrolysis of GTP bound to Arfs [11,89]. In humans, Arf GAP proteins are encoded by 31 genes and some have multiple splice variants. Based on sequence homology and the overall domain structure, Arf GAPs have been divided into ten groups (Figure 1). In six groups, the Arf GAP domain is at the N-terminus, followed by distinct domains in the C-terminal region that place them into specific groups. Four groups have a more complex domain structure. In addition to a tandem of PH, Arf GAP and Ankyrin repeat domains responsible for the catalytic GAP activity, the latter four groups also contain multiple domains capable of binding to lipids, proteins and catalyzing RhoA•GTP hydrolysis. These Arf GAPs are named after the domain unique to the group: ASAPs for the SH3 domain present in ASAP1/2, ACAPs for the coiled-coil (BAR domain), ARAPs for the Rho GAP domain, and AGAPs for the GTP-binding protein-like domain. 

## 5. Arf GAPs that Regulate Circular Dorsal Ruffles (CDRs)

Five Arf GAPs including ASAP1/3, ACAP1/2 and ARAP1 localize to CDRs, and four of them have been shown to affect the formation or the ring size of CDRs [27,34,35,42]. Overexpression of ASAP1 suppressed and ASAP1 knockdown increased CDR formation [27,28]. This effect of ASAP1 on CDRs depends on GAP activity and the BAR (Bin/Amphiphysin/Rvs) domain. The BAR domain of ASAP1 binds to non-muscle myosin 2A (NM2A) and NM2A knockdown produced a similar stimulatory effect on CDRs to ASAP1 knockdown. Moreover, the overexpression of NM2A rescued the ASAP1 knockdown phenotype in CDRs [27]. Together, these results indicate that NM2A mediates the ASAP1 control of CDRs. Like ASAP1-overexpressing cells, cells overexpressing ACAP1 or ACAP2 also exhibited fewer CDRs, but the GAP activity of ACAP1 and ACAP2 only partly accounts for their inhibition on CDR formation and the specific mechanisms have not been explored.

ARAP1 localizes to the inside of the F-actin ring of CDRs and controls the ring size of CDRs [42]. The effect of ARAP1 on CDR formation opposed that of ASAP1 even though both functioned as Arf1/5 GAPs and exerted their effects on CDRs in a GAP-dependent fashion. Cells with reduced ARAP1 expression formed fewer and smaller CDRs, while the overexpression of ARAP1 led to larger CDRs. This effect of ARAP1 on CDRs depends on the Arf GAP activity but not on the Rho GAP activity. In contrast, the efficient targeting of ARAP1 to CDRs requires three of its PH domains (PH3~5), Rho GAP domain and RA domain at the C-terminus but not the Arf GAP domain. The precise role of Arf GAP activity in controlling the formation and size of CDRs for each of the CDR-associated Arf GAPs remains elusive and likely to be distinct for each Arf GAP. Both constitutively active (CA) mutants of Arf1 and Arf6 inhibited the CDR formation whereas CA Arf5 had no effect [42], indicating that low levels of Arf1•GTP and Arf6•GTP are required for CDR formation. This also suggests that ASAP1 and ACAP1/2 must exert their effects on CDRs by mechanisms in addition to inactivating Arf1 and Arf6. In the case of ASAP1, the regulation of NM2A activity is likely important and could depend on the GAP activity of ASAP1 [90]. ACAP1 and ACAP2 also contain BAR domains, so it is possible that they might also bind and regulate NM2A like ASAP1. Recently, Rab35 has been shown to be required for CDR formation by regulating PI3K signaling [91]. ACAP2 binds Rab35•GTP and mediates the effect of Rab35 on phagocytosis and neurite outgrowth [9,10,36,38,92]. To date, all studies on ACAP2’s functions with Rab35 have indicated the need for the cycling of Arf6 GTP/GDP, which may explain the contradictory results that both ACAP2 overexpression and CA Arf6 inhibited CDR formation since both treatments disrupted the cycles of Arf activation and inactivation. The dominant negative (DN) form of Arf1 increased the ring size but prevented ring closure, indicating Arf1•GTP is needed for ring constriction. In addition to low Arf1 and Arf6 activity, the conversion of PI(3,4,5)P3 to PI(3,4)P2 by the PIP3-5 phosphatase, SHIP2, is also necessary for CDR formation [93]. One proposed model posits that, upon PDGF stimulation, PI3K is activated to produce PI(3,4,5)P3, which stimulates the ARAP1 Arf GAP activity, lowering Arf1•GTP levels, and it also recruits SH3YL1 (SH3 domain containing Ysc84-like 1) and the associated SHIP2 to convert PI(3,4,5)P3 to PI(3,4)P2. The two events lead to the formation of CDRs. As the PI(3,4,5)P3 levels decrease, ARAP1 is gradually deactivated, leading to increased Arf1•GTP and the closure of the CDR ring. However, while overexpression of the DN mutants of Arf1 or Arf5 recapitulated the larger CDR ring phenotype induced by ARAP1 overexpression, these mutants failed to rescue the reduced numbers and size of CDRs caused by the ARAP1 knockdown. We speculate that, like ASAP1 and ACAP1/2, downstream targets of ARAP1 besides Arf1 or Arf5 must be involved, and the Arf GAP activity may regulate the ARAP1 interactions with these targets. 

## 6. Arf GAPs that Regulate Podosomes and Invadopodia

Five Arf GAPs have been shown to associate with podosomes or invadopodia: ASAP1 with podosomes induced by active Src in fibroblasts and invadopodia in cancer cells [29]; ARAP1, GIT1 and GIT2 with podosome belts in osteoclasts [41,63]; and ARAP3 with podosome-like structures in fibroblasts plated on a fluid substrate [50]. ASAP1 knockdown suppressed podosome formation. GAP activity is not required for the effect of ASAP1 on podosome formation. Instead, the SH3 domain and Src-mediated phosphorylation of a tyrosine residue are required with additional contribution from the BAR domain [29]. To digest bone, osteoclasts form a sealing zone by coalescing podosomes into a belt. ARAP1 expression increased during osteoclastogenesis. In matured osteoclasts, ARAP1 localized to podosomes and sealing zones in which lysosomal enzymes are secreted to digest the bone matrix [41]. ARAP1 knockdown inhibited the formation of the podosome belts and bone resorption. Cells with reduced ARAP1 formed fewer and very small, static sealing zone structures. ARAP1 isoform2 containing PH3 and 4, Rho GAP, RA and PH5 domain, still targeted to the podosome belt and rescued the defect in sealing zone formation, suggesting that the Rho GAP domain regulates the formation and dynamics of podosome belts [41]. This is different from ARAP1 control of CDRs, wherein Arf GAP instead of Rho GAP activity is required, even though it appeared that the C-terminal half of ARAP1 is the targeting domain in both cases. GIT1 knockout and GIT2 knockdown prevented podosome belt formation [53,63]. Moreover, GIT1 knockout mice exhibited increased bone mass consistent with defective bone resorption in GIT1 knockout osteoclasts. It is unclear whether the GAP activity of GIT1/2 is needed for this function [53]. 

The mechanisms by which ASAP1, ARAP1, ARAP3, GIT1 and GIT2 facilitate podosome formation await further investigation. Several lines of evidence supported the idea that the RhoA-NM2A pathway is likely involved and that podosome-associated Arf GAPs act as Arf effectors. First, the Arf GAP activity of ASAP1 and ARAP1 is dispensable for their effects on podosome formation. Second, Arf1 was required for the formation of podosomes induced by various stimuli such as TGFβ1 or PMA (Phorbol 12-myristate 13-acetate) treatment in macrophage-like THP1 cells or active Src in fibroblasts [94]. Blocking Arf1 activation by knocking down ARNO also disrupted podosome formation, suggesting that Arf1•GTP but not the GTP hydrolysis on Arf1 is required. Consistent with this idea, CA Arf1 induced the formation of actin-rich puncta that were capable of degrading ECM but were more motile and dynamic than the mature podosomes. Third, Rho family proteins play important roles in podosome formation. High Rac and Cdc42 activity promote whereas high RhoA activity inhibits podosome formation [95]. Interestingly, inhibiting Arf1/6 activity by SecinH3 increased RhoA•GTP and inhibited podosome formation in an NM2A-dependent manner. Finally, ASAP1, ARAP1 and ARAP3 could potentially regulate NM2A or RhoA activity via the BAR domain and RhoGAP domain respectively, which are responsible for at least part of their effect on podosomes. The BAR domain of ASAP1 directly binds NM2A. Additionally, another BAR domain-binding protein, GEFH1, is a RhoGEF that attenuated podosome formation [96]. In concert, these results are compatible with the hypothesis that ASAP1 and ARAP1 function as Arf1 effectors to promote the formation or maintenance of podosomes by downregulating RhoA-NM2A activity.

## 7. Arf GAPs in Motility-Related Structures: Lamellipodia, Stress Fibers and Focal Adhesions

Lamellipodia, FAs and stress fibers mediate key steps in the cell motility cycle: protrusion at the leading edge, adhesion and retraction of the trailing end. The dynamics of the three structures are coordinately regulated to meet the spatial and temporal need for cell migration. Unsurprisingly, Arf GAPs that regulate these structures often affect cell migration with effects on more than one of the structures. Of these Arf GAPs, ASAP1, GIT1, GIT2 and ARAP1-3 are the most extensively studied and thus will be the focus of our discussion. A more comprehensive view of all the Arf GAPs involved has been covered in a recent review [97].

### 7.1. ASAP1

ASAP1, GIT1, GIT2 and ARAP2 localize to and regulate FAs. ASAP1 targets to FAs by interaction with FAK via the SH3 domain and with Crk via the proline-rich domain [31,32]. ASAP1 knockdown decreased the numbers of mature FAs and disrupted stress fibers with reduced colocalization of NM2A and F-actin [27]. The maturation of FAs from the small nascent adhesions requires tension in adhesions created by the contractile stress fibers. Together with the finding of direct interaction between ASAP1 and NM2A, we propose a model in which the adhesion-associated ASAP1 may stimulate NM2A activity and facilitate the assembly of stress fibers, resulting in FA maturation [90]. It is not clear whether the Arf GAP activity of ASAP1 plays a role in the formation of stress fibers or FAs. 

### 7.2. GIT1 and GIT2

GIT1 has been reported to promote and inhibit actin-driven membrane protrusions by increasing and decreasing GTP bound forms of Rac1 and/or Cdc42 respectively [97,98]. Some of the discrepancies can be explained by the use of an N-terminal epitope on GIT1, which completely abrogates GAP activity [98], therefore when overexpressed, may act as a dominant negative protein to disrupt GIT1 normal function. Taking this into account, it becomes clear that the effects of GIT1 and GIT2 on lamellipodia, FAs or motility depend on the cell type. In endothelial cells, both GITs appear to promote lamellipodia formation and FA disassembly. On the contrary, in most epithelial cells and fibroblasts, GITs inhibit lamellipodia formation and stabilize FAs by inactivating Rac1 although the specific mechanisms vary between GIT1 and GIT2. The distinct effects may be explained by the difference in the Rac1/Cdc42 exchange factors involved or the role of Arf6 GAP activity under the control of various stimuli in different types of cells.

In endothelial cells and non-small-cell lung cancers (NSCLCs), GIT1 functions with PIX (PAK-interacting exchange factor), an exchange factor for Rac1 and Cdc42, to positively regulate Rac1 and Cdc42 activity, and cortactin, an F-actin binding and bundling protein, to promote membrane protrusions and cell migration [56,99]. The role of Arf GAP activity was not examined in these studies. GIT1 knockdown decreased Rac1•GTP and Cdc42•GTP levels and migration/invasion of NSCLC cells [99]. Overexpression of Rac1 and Cdc42 CA mutants rescued the reduced cell migration and invasion caused by GIT1 knockdown. Similarly, GIT1 promoted lamellipodia formation and the directional migration of endothelial cells towards the vascular endothelial growth factor (VEGF) [56]. The mechanism appears to be that GIT1 promotes cortactin targeting to lamellipodia via the activation of Rac1/Cdc42 and ERK (extracellular signal-regulated kinase) pathways. GIT1 binds cortactin and PIX via its spa homology domain (SHD). Cortactin promotes actin polymerization and organization by binding to Arp2/3 complex, F-actin and N-WASP, mediating Rac1/Cdc42-induced actin remodeling in the lamellipodia [100]. Phosphorylation of cortactin also regulates its effect on actin filaments. For example, serine 405 and 418 phosphorylation by ERK1/2 facilitate cortactin binding to N-WASP and Arp2/3-dependent actin polymerization [100]. In endothelial cells, GIT1 knockdown reduced Rac1•GTP and Cdc42•GTP levels, and consequently VEGF-induced ERK activation, thereby inhibiting lamellipodia formation and cortactin localization to the lamellipodia. The effect of GIT1 on ERK and pS405 cortactin depended on the PIX and cortactin-binding domain, SHD, and phosphorylated cortactin-mimicking mutant (S405D/S418D) rescued GIT1 knockdown-reduced migration. Together, these results support the idea that GIT1 promotes lamellipodia formation by binding to PIX via SHD, resulting in Rac1 and Cdc42 activation. Active Rac1 and Cdc42 can then stimulate actin polymerization by activating actin nucleation-promoting factors like WAVE or N-WASP or activating PAK to activate ERK and cortactin. GIT2 was also reported to promote endothelial cell migration in response to CXCR2 by function with another Rac GEF, Vav2 [101]. Mechanistically, GIT2 appeared to stimulate Vav2, leading to Rac activation and increased migration induced by CXCR2.

**Figure 2:**
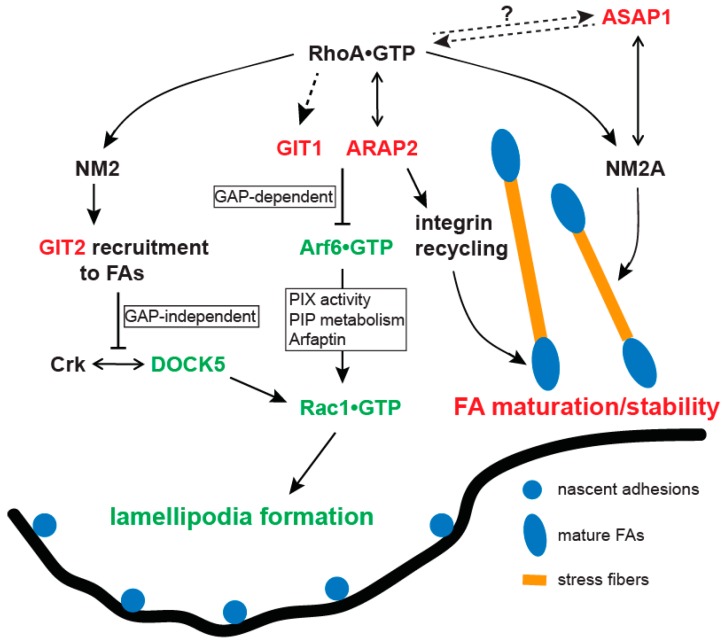
The roles of ASAP1, GITs and ARAP2 in the regulation of actin structures involved in lamellipodia-dependent cell migration. The cartoon illustrates the pathways that ASAP1, GITs and ARAP2 control to modulate the antagonistic relationship between lamellipodia formation and stress fiber formation/focal adhesion (FA) maturation. Arrows indicate positive/stimulatory regulation while ⊥ indicates negative/inhibitory regulation. Double arrows indicate binding. Dash lines denote possible regulation and the question mark denotes ASAP1 may function upstream or downstream from RhoA.

In fibroblasts and most epithelial cells, GIT1 and GIT2 inhibit lamellipodial protrusions and stabilize FAs by reducing Rac•GTP levels [60,61,102]. Overexpression of the recombinant GIT1 with an N-terminal tag (GAP-deficient) destabilized FAs and promoted membrane protrusions in epithelial cells including HeLa, COS7 and CHO cells as well as in REF52 fibroblasts [58,98,103], suggesting that the GAP activity of GIT1 normally functions to limit membrane protrusions and FA turnover. Consistent with this interpretation, in migrating CHO cells that stably express α4 integrins [102], GIT1 was restricted to the sides and rear of the cells by binding to paxillin because paxillin cannot bind to the phosphorylated α4 integrin in the leading edge of the cells. As a result, GIT1 inhibited membrane protrusions at the sides and rear of the cells by functioning as an Arf6 GAP to reduce Arf6•GTP and consequently Rac1•GTP levels (Figure 2). Although GIT1 associates with PIX, whether PIX mediated the change in Rac1 activity downstream from Arf6 was not investigated in this work. There are several ways that Arf6 can affect Rac1 activity. Arf6 can activate phosphatidylinositol 4 phosphate 5 kinase (PI4P5K) to produce PI(4,5)P2 [104], a precursor for PI(3,4,5)P3 that can activate several Rac1 GEFs including Vav2, Tiam1 and PIX. Furthermore, Arf6•GTP can displace Rac1 from Arfaptin, thereby releasing Rac1 for activation [105]. Arf6•GTP can also influence the binding of PIX with GIT1, as well as the Rac1GEF activity of PIX. GIT1 in the complex of paxillin-α4 integrin may modify GIT1 Arf6 GAP activity or alter the effect of GIT1 on the associated Rac1GEF to elicit the opposite effect on membrane protrusions described in other studies with endothelial cells.

GIT2 also inhibits membrane protrusions and stabilizes FAs by reducing Rac1 activity in epithelial cells including HeLa, MCF10A and MDA-MB-231 [60,61]. However, unlike the case with GIT1 in α4 integrin-associated inhibition of protrusions, GIT2’s effects are independent of Arf6. GIT2 localizes to mature FAs in a manner that is dependent on Myosin 2-induced contractility [60,61,106]. Inhibition of Myosin 2 contractility by Blebbistatin or ROCK inhibitor prevented GIT2 localization to FAs. Mechanistically, GIT2 suppresses the Rac1 exchange factor DOCK5 [107] and membrane protrusions by inhibiting the DOCK5 interaction with Crk [60,61]. GIT2 knockdown promoted lamellipodial protrusions and FA turnover, which was blocked by DOCK5 knockdown but not PIX knockdown. Correspondingly, DOCK5 knockdown inhibited membrane protrusion and stabilized FAs. GIT2 knockdown promoted the DOCK5 interaction with Crk and permitted colocalization of DOCK5 with paxillin, indicating that GIT2 limited the targeting of DOCK5 and the consequent Rac1 activation at adhesions to stabilize FAs. Moreover, overexpressing the Crk-binding domain of DOCK5 abolished the enhanced membrane protrusions by GIT2 knockdown. Taken together, GIT2 represents a new mediator of the antagonism between Rho and Rac. Rho-dependent activation of Myosin 2 targets GIT2 to FAs where GIT2 induces the dissociation of DOCK5 from Crk, leading to Rac1 inactivation and FA stabilization and maturation (Figure 2). 

### 7.3. ARAP2

ARAP2 may also mediate the Rho-Rac antagonism via an Arf6 GAP-dependent mechanism, distinct from GIT2. Through its catalytically-inactive Rho GAP domain, ARAP2 can bind RhoA•GTP [47]. ARAP2 promotes the formation of stress fibers and FAs depending on its Arf6 GAP and RhoA binding activities [45,47]. A decrease in Arf6•GTP levels by ARAP2 leads to a decrease in Rac1•GTP, which is required for the effect of ARAP2 on stress fibers and FAs. The inhibition of Arf6 activity by a DN mutant of Arf6 restored the number of stress fibers and mature FAs in ARAP2 knockdown cells. However, this inactive Arf6 form did not increase the number or size of FAs to the same extent as ARAP2 overexpression. Other ARAP2-dependent functions like directing the endocytic trafficking of integrins may contribute to the full effect of ARAP2 on these structures [46,48]. 

Collectively, the studies on ASAP1, GIT1, GIT2 and ARAP2 in epithelial cells and fibroblasts are most consistent with a model in which ASAP1 associates with nascent adhesions where it interacts with and promotes NM2A activity to assemble stress fibers, generating contractility necessary for FA maturation. GIT1/2 and ARAP2 are recruited to FAs in response to Rho and Myosin 2 activation to inhibit Rac1, leading to FA maturation (Figure 2). GIT1 and ARAP2 inhibit Rac1 by inactivating Arf6 via their GAP activity whereas GIT2 inhibits Rac1 by suppressing DOCK5 interaction with Crk, independent of Arf6. Finally, ARAP2 also maintains the stability of FAs at least in part by controlling the recycling of integrins [46,48].

### 7.4. ARAP1 and ARAP3

In contrast to ARAP2, ARAP1 and ARAP3 function upstream from RhoA to suppress the formation of stress fibers, actin structures known to be induced by RhoA activation. Both ARAP1 and ARAP3 contain a catalytically active Rho GAP domain. ARAP1 has been reported to have Rho GAP activity against RhoA and Cdc42. ARAP3 was reported to prefer RhoA as a substrate [44,108]. Consistent with and dependent on their Rho GAP activity to reduce RhoA•GTP levels, the overexpression of ARAP1 or ARAP3 resulted in fewer stress fibers in cells [44,109,110]. ARAP1, as well as ARAP3, localize to the cell periphery and affect the associated actin structures in addition to stress fibers, including filopodia and lamellipodia [44,52,108,109]. Filopodia are finger-like membrane projections made of bundles of actin filaments emanating from the lamellipodia to probe the extracellular environment during cell migration [2]. Overexpression of ARAP1 in fibroblasts promoted filopodia formation dependent on elevated Cdc42•GTP [44]. The effect was independent of ARAP1 Rho GAP activity and instead required its Arf GAP activity. Both overexpression and reduced expression of ARAP3 inhibited lamellipodia formation, an effect dependent on the Arf GAP and Rho GAP activity [52,108,109]. In summary, ARAPs affect actin structures via both Rho GAP-dependent and -independent mechanisms. In cases where the Rho GAP activity is dispensable, Arf GAP activity is often required. In vitro, PIP3 stimulated the Arf GAP activity of ARAPs but had no effect on the Rho GAP activity of ARAP1 and 3. For ARAP3, Rap•GTP binding to the RA domain activated the Rho GAP activity, a step that is also necessary for the effect of ARAP3 on lamellipodia [44,108]. Therefore, PIP3 and Rap signaling could converge on ARAPs to modulate the activity of Rho family proteins by Arf inactivation-dependent or -independent mechanisms. The mechanisms by which the Arf GAP activity regulates Rho proteins remain elusive. The Arf6 GAP, ARAP2, may inhibit Rac1 by lowering Arf6•GTP levels in a manner similar to that postulated for GIT1 inhibition of Rac1 when associated with α4 integrin: Arf6•GTP can activate Rac1 by changing the phosphoinositide metabolism, the activity of Rac1 GEF or Rac1 GDI (guanine nucleotide dissociation inhibitor). 

## 8. Arf GAPs in Engulfment of Pathogens and Apoptotic Cells

Many Arf GAPs were shown to affect the internalization of pathogens and apoptotic cells, playing both positive and negative roles in these processes. Phagocytosis of bacterial pathogens or apoptotic cells is initiated by the specific “eat me” ligand-receptor pairs on cells. For example, IgG-bound bacteria recognized by the opsonic receptor FcγR on macrophages, β-glucan from yeast cell walls (zymosan) recognized by the pattern-recognition receptors on macrophages, and phosphatidylserine on apoptotic cells by the apoptotic corpse receptor on fibroblasts [19,22]. Macropinocytosis is a form of endocytosis for the nonspecific bulk uptake of extracellular fluid. It occurs in various forms in many cell types and is often induced by growth factors. CDRs, for example, are one type of macropinocytic ruffles. Many viruses and bacteria hijack macropinocytosis to enter host cells utilizing signaling events in the activation of the growth factor receptors [23,24]. Actin polymerization is required for both phagocytosis and macropinocytosis. 

One group of Arf GAPs including ASAP1/2, ACAP1/2 and ADAP1 functioned to facilitate Arf GTP/GDP cycles for the internalization of pathogens into phagocytic and non-phagocytic cells. Both ASAP2 and ACAP2 are involved in FcγR-mediated phagocytosis [10,12,33]. ACAP2 also affected zymosan-induced phagocytosis [9]. ASAP2 colocalized with F-actin at the phagocytic cup. Overexpression of ASAP2, but not the GAP-inactive mutant, reduced the accumulation of F-actin at the phagocytic cup and phagocytosis [12]. ASAP2 may function as an Arf6 GAP in cells [111] although, in vitro, ASAP2 preferentially uses Arf1 and Arf5 as substrates [112]. Interestingly, DN or CA mutant of Arf6 also inhibited the accumulation of F-actin beneath the IgG-coated particles and FcγR-mediated phagocytosis like ASAP2 overexpression, suggesting that the cycling of Arf6 between GTP- and GDP bound forms is critical for actin remodeling and phagocytosis [80] and that ASAP2 facilitates this Arf6 GTP/GDP cycling through its GAP activity. Recently, ASAP2 was found to interact with selenoprotein K (Selk), a palmitoylation cofactor required for FcγR-mediated phagocytosis. SelK-dependent palmitoylation of ASAP2 resulted in the cleavage of ASAP2 within the BAR domain by Calpain2, and consequently, the dissociation of ASAP2 from the phagocytic cup [33]. Together, it appeared that the dynamic association and dissociation of ASAP2 with the phagocytic cup, which can be controlled by Selk, resulting in oscillation of Arf6•GTP hydrolysis, is essential for FcγR-mediated phagocytosis. Another Arf6 GAP, ACAP2, seemed to function in a similar way during FcγR- or zymosan-induced phagocytosis [9,10,12]. The overexpression of ACAP2 also inhibited phagocytosis dependent on its GAP activity. Different from ASAP2, the association of ACAP2 with the phagocytic membrane was regulated by Rab35. Rab35•GTP recruited ACAP2 to the phagocytic membrane. CA and DN Rab35 inhibited phagocytosis, supporting the idea that cycles of Arf6 activation and inactivation can be achieved by controlling ACAP2 on and off the phagocytic membrane by Rab35 GTP/GDP cycles. In this model, the role of GAP activity of ASAP2 or ACAP2 was not to lower the Arf6•GTP levels *per se*, but to ensure repeated Arf6 activation and inactivation, which is essential for phagocytosis. As yet untested, one prediction of this hypothesis is that the knockdown of ASAP2 or ACAP2 should inhibit phagocytosis just as is observed for the overexpression of ASAP2 or ACAP2 because either will inhibit Arf6 cycles between the active and inactive forms. Another prediction is that a fast cycling mutant of Arf6 should rescue the reduced phagocytosis in ASAP2 or ACAP2 knockdown macrophages.

**Figure 3:**
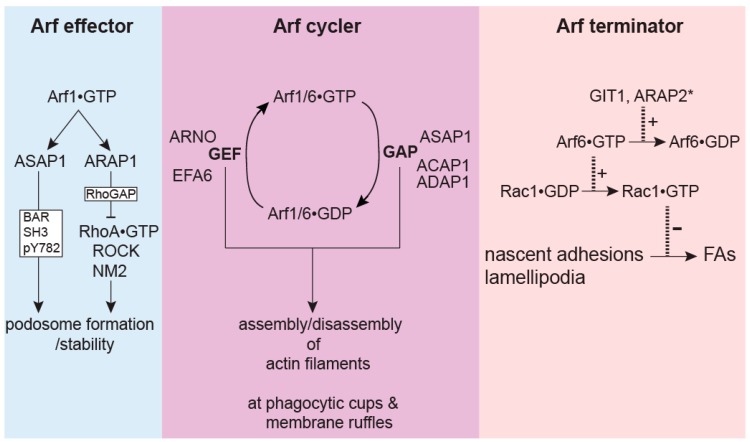
The modes of action that Arf GAPs take to regulate actin and related functions. Arf GAPs regulate actin-based structures and functions by acting like (1) Arf effectors that bind Arf•GTP, propagate and promote Arf functions independent of GAP activity; (2) Arf cyclers that, by working with Arf GEFs, facilitate the cycling of Arfs between GTP and GDP bound states to aid dynamic assembly and disassembly of actin structures, and (3) Arf terminators that reduce Arf•GTP levels and terminate consequent signaling for actin remodeling. Examples for each mode of action are shown. ARAP2* denotes that parts of ARAP2 function in focal adhesions (FAs) are mediated by other mechanisms. + and − mean “promoting” and “inhibiting” respectively.

These ideas were tested in studies on the role of Arf GAPs in the entry of *Salmonella* into cells [26]. Similar to FcγR- or zymosan-induced phagocytosis, *Salmonella* invasion of the non-phagocytic intestinal epithelial cells also requires the remodeling of the actin cytoskeleton. ACAP1, ADAP1 and ASAP1 (but not ArfGAP1, GIT1 or ARAP3) localize to *Salmonella*-induced membrane ruffles driven by actin polymerization. Both Arf1•GTP and Arf6•GTP are required for activating the WAVE regulatory complex (WRC) to stimulate actin polymerization at these ruffles. Interestingly, both the overexpression and knockdown of ACAP1, ADAP1 and ASAP1 inhibited the *Salmonella* invasion. Consistent with this result, both CA and DN Arf1 or Arf6 inhibited the *Salmonella* invasion. Arf GAP activity is required for the inhibitory effect on *Salmonella* invasion as GAP dead mutants of these Arf GAPs no longer had an effect on the invasion. Fast recycling mutants of Arf1 and Arf6 rescued the decreased invasion in ASAP1 knockdown and ADAP1 knockdown cells respectively. The overexpression of the Arf1 GEF, ARNO, and Arf6 GEF, EFA6, abolished the inhibition on invasion caused by the overexpression of ASAP1, and ACAP1 and ADAP1 respectively, supporting the idea that specific Arf GAPs and Arf GEFs work as pairs to ensure cycling of Arfs between the GTP and GDP bound forms and the consequent repeated assembly of actin-based ruffles necessary for multiple rounds of *Salmonella* invasion (Figure 3).

Another group of Arf GAPs including ARAP2 and ArfGAP1 acted as terminators of Arf signaling to affect the pathogen uptake and phagocytosis of apoptotic cells. Through an siRNA screening, ARAP2 was identified to be important for *Listeria monocytogenes* entry into epithelial cells [49]. *Listeria* binds and activates the Met receptor on the host cell to induce rearrangement of the actin cytoskeleton necessary for its internalization into non-phagocytic cells [113]. ARAP2 knockdown impaired the *Listeria*-induced recruitment of F-actin and entry into cells. Arf GAP-deficient and Rho binding mutants both decreased pathogen entry compared with wild-type ARAP2. Arf6 CA mimicked ARAP2 siRNA-decreased entry and Arf6 knockdown reversed the effect of the GAP-deficient but not the Rho-binding mutant on *Listeria* entry. These results support a role of ARAP2 acting downstream from the Met receptor to promote actin remodeling, which is exploited by *Listeria*. *Listeria* induces Met activation and subsequently PI3K activation, producing PI(3,4,5)P3. PI(3,4,5)P3 activates the Arf6 GAP activity of ARAP2 to reduce Arf6•GTP levels, leading to actin remodeling necessary for *Listeria* engulfment. 

ArfGAP1 is involved in the entry of another pathogen, *Mycobacterium tuberculosis* (Mtb), into epithelial cells [25]. Remodeling of the actin cytoskeleton is important for the uptake of Mtb by epithelial cells. Mtb induced Arf1 and PLD1 activation downstream of muscarinic receptor 3 (M3R), leading to the reorganization of cortical actin and formation of actin stress fibers that facilitate mycobacterial entry. ArfGAP1 inhibited this bacterial entry by reducing Arf1•GTP and PLD1 activity and the consequent actin remodeling. The effect of inhibiting bacterial entry may be GAP activity-dependent because QS11, a putative inhibitor of ArfGAP1, mimicked the increased mycobacterial entry seen with the ArfGAP1 knockdown [25,114]. This effect of ArfGAP1 is specific for mycobacterial uptake in epithelial cells as ArfGAP1 knockdown did not affect the uptake of *Shigella flexneri* or *Yersinia pseudotuberculosis*. Additionally, ArfGAP1 only inhibited mycobacterial entry in epithelial cells but not in monocytes or macrophages, suggesting that ArfGAP1 does not regulate phagocytosis, another mechanism of pathogen uptake. The precise mechanism by which ArfGAP1 regulates the actin rearrangement for Mtb entry remains to be determined. ArfGAP1 knockdown reduced cortical F-actin and increased actin stress fiber formation. Cytochalasin D inhibited mycobacterial uptake and abolished ArfGAP1 knockdown-increased uptake. Some pathogens manipulate the activity of RhoA, Rac1 or Cdc42 to reorganize the actin cytoskeleton to enter cells. However, mycobacterial entry did not appear to involve these three Rho proteins; knockdown of RhoA, Rac1 or Cdc42 did not reverse the effect of ArfGAP1 knockdown on Mtb uptake. It is still possible that other members in the Rho family may play a role, or Rac1 and Cdc42 play redundant roles.

GIT1 is involved in phagocytosis of apoptotic cells [59]. G protein-coupled receptor kinase 6 (GRK6) stimulated phagocytosis of apoptotic thymocytes by NIH3T3 fibroblasts depending on Rac activity. DN Rac abolished GRK6-stimulated phagocytosis. GIT1 interaction with GRK6 is critical for this function. GRK6-induced Rac activation and phagocytosis were blocked by a GIT1 mutant deficient for binding to GRK6 (deletion of the coiled-coil domain between SHD and PBS2). GIT1 might contribute to Rac activation via its interaction with the Rac GEF, PIX.

The mechanisms for coupling Arf6 GTP/GDP cycles to actin reorganization supporting the formation of phagocytic cups and effective phagocytosis is not completely understood. PI(4,5)P2 is likely involved. PI(4,5)P2 binds and regulates the activity of many actin-binding proteins such as N-WASP/WAVE, cofilin, profilin and gelsolin, which control the polymerization and depolymerization of actin filaments [1,3]. PI(4,5)P2 also binds and activates proteins that link actin filaments to the plasma membranes, such as ERM (ezrin/radixin/moesin) proteins and talin. PI(4,5)P2 can be produced by the Arf6 effector, PI4P5K. Therefore, it is plausible that Arf6 GTP/GDP cycles allow oscillations of PI(4,5)P2 levels to dynamically assemble actin filaments at the plasma membrane, which is necessary for forming the phagocytic cup with protrusions changing in size to engulf pathogens. 

## 9. Arf GAPs that Regulate Neurite Outgrowth or Neuron Migration

Neurite outgrowth depends on growth cones whose morphology, molecular composition and regulation are reminiscent of those in the lamellipodia [115,116]. Consistent with the roles of GIT1 and GIT2 in controlling actin networks and adhesion dynamics within the lamellipodia, they are implicated in neurite branching and exerted their effects, at least in part, by interaction with PIX. GIT1 knockdown reduced neurite branching in GABA-ergic interneurons [55]. The interaction with PIX and paxillin via the SHD and PBS2 domain of GIT1 are required for the effect on neurite branching. Knockout of Rac1 and Rac3 in interneurons also impaired neurite branching. The growth cone, similar to the lamellipodia of a migrating cell, is driven by actin polymerization and requires the turnover of the adhesions [116]. GIT1 knockdown decreased the growth cone area but increased the proportion of fan-shaped growth cones. In hippocampal neurons, the knockdown of GIT2 but not GIT1 reduced neurite branching in hippocampal neurons with defects similar to those by αPIX knockdown [62].

ACAP2 functions with Rab35 to promote nerve growth factor (NGF)-induced neurite outgrowth [36,37,38]. ACAP2 appeared to act downstream from Rab35 to negatively regulate Arf6 activity [92]. This is different from facilitating Arf6 GTP/GDP cycling by ACAP2/Rab35 in phagocytosis. CA Rab35 enhanced whereas DN Rab35 inhibited NGF-induced neurite outgrowth in PC12 cells [37]. ACAP2 functions as a Rab35 effector [37], binding to Rab35•GTP to decrease Arf6•GTP levels. The Arf6 GAP activity is required for ACAP2-dependent neurite outgrowth and CA Arf6 inhibited neurite outgrowth. The Rab35 and ACAP2 interaction was essential for their effects on neurite outgrowth as neither of the binding-deficient mutants, Rab35(T76S/T81A) and ACAP2(N610A/N691A) rescued the suppressed neurite outgrowth in PC12 knockdown cells [38]. 

ACAP3 functions as an Arf6 GAP, measured by a pull-down assay, showed strong expression in the cortex, hippocampus and cerebellum and preferentially localized to the tip of the growth cone, suggesting a role in axonal outgrowth [39]. Different than ACAP2 that functions simply to inactivate Arf6, ACAP3 ensured the cycling of Arf6 between active and inactive forms to aid neuronal functions. ACAP3 promoted neurite outgrowth in hippocampal neurons. Accordingly, ACAP3 knockdown shortened the length of neurites without affecting the numbers of neurites, which can be reversed by wild-type ACAP3 but not the GAP-deficient mutant of ACAP3. Like ACAP3 knockdown, Arf6 knockdown or knockout inhibited neurite outgrowth. Interestingly, the ACAP3 knockdown phenotype can be rescued by the fast cycling mutant of Arf6 [117] but not the wild-type, DN or CA mutant of Arf6 [39]. This result parallels the involvement of Arf GAPs in *Salmonella* entry, wherein impaired pathogen entry due to Arf GAP depletion was rescued by fast cycling mutants of Arfs [26]. In addition, consistent with the abundant expression of ACAP3 in the cerebral cortex, ACAP3 knockdown blocked the radial migration of neurons in the cortex [40]. This defect was also Arf GAP dependent, as the GAP-deficient mutant of ACAP3 failed to rescue it. Neurons with reduced ACAP3 exhibited abnormal morphology with multipolar or no processes in contrast to the uni/bipolar morphology of control cells. Overexpression of DN or CA mutant of Arf6 impaired neuronal migration in the cerebral cortex [118]. 

## 10. Conclusions and Future Perspectives

As discussed in this review, Arf GAPs have emerged as important regulators of the actin cytoskeleton. There are several noticeable features for the action of Arf GAPs in actin remodeling. One feature is the cross-talk with Rho family proteins with effects on RhoA, Rac1 and Cdc42. Rho proteins may function downstream of Arf GAPs and Arfs. For example, Rac1 can be regulated by the Rac1 GEF PIX binding to GIT1 or by signaling downstream from Arf6. ARAP1 and 3 can inhibit RhoA through their Rho GAP activity. Rho family proteins may also function upstream or in parallel with Arf GAPs and Arfs. For example, GIT2 and ARAP2 function downstream of RhoA to suppress Arf6 activity. NM2A functions downstream from RhoA and ASAP1; cortactin functions downstream from GIT2 and Rac1/Cdc42, suggesting that Arf GAPs act in parallel with Rho proteins to regulate these actin-binding proteins. Second, the targeting of Arf GAPs is usually mediated by a domain outside of the Arf GAP domain (Table 1), and not by binding to PIPs via the PH domains. This is consistent with the idea that PIP-binding to the PH domain mainly functions to stimulate Arf GAP activity, not to recruit GAPs to the membranes [119,120]. Finally, as summarized in Figure 3, the effects of Arf GAPs on actin remodeling extend beyond the negative regulation of Arfs. 

Arf GAPs are important for cellular functions involving distortion of the plasma membrane, which is supported by changes in actin polymerization or organization. While we focus on the regulation of actin remodeling by Arf GAPs and the implication in these cellular functions, it is important to recognize that the remodeling of the actin cytoskeleton is integrated with the vesicular trafficking and both are controlled by Arf GAPs. For example, the extension of membranes during phagocytosis, lamellipodial protrusion or the formation of the highly convoluted CDRs and podosomes, requires a supply of the membrane from the endocytic or secretory pathway [121]. At the same time, actin polymerization must be coordinately regulated in the juxtamembrane region to give mechanical support for the added membranes. For podosomes and invadopodia that digest bone or ECM, membrane trafficking must be coordinated with the assembly of the adhesive actin structures in order to deliver metalloproteases or lysosomal proteases to specific locations on the plasma membrane [41]. Thus, Arf GAPs represent a class of important regulators that function with Arfs and Rho proteins, to coordinate the changes in the actin cytoskeleton and membrane traffic. With multiple Arf GAPs acting at the same sites, one challenging but important question is how cells coordinate their relative activities to control machinery for cytoskeleton and membrane traffic.

## Figures and Tables

**Table 1:** Arf GAP-dependent actin structures and associated functions.

Arf GAP	Domains Required for Function	Actin Structures Affected	Function	Reference
ArfGAP1	Arf GAP	Stress fibers	Limits stress fiber formation to restrict mycobacterial entry	[25]
ADAP1	Arf GAP	Actin-based membrane ruffles	Facilitates Arf6 GTP/GDP cycles and actin remodeling necessary for *Salmonella* invasion	[26]
ASAP1	Arf GAP, BAR	CDRs	Inhibits CDR formation through NM2A/GAP activity	[27,28]
	SH3, Src-mediated phosphorylation, BAR, (Arf GAP-independent)	Podosomes and Invadopodia	Promotes podosome formation in fibroblasts and invadopodia in cancer cells	[29]
	Arf GAP	N.D.	Promotes migration and invasion of MDA-MB-231 cells	[30]
	N.D. (Not-determined)	Stress fibers, focal adhesions (FAs)	Increases mature FAs and assembly of stress fibers	[27]
	Arf GAP (partly)		Inhibits cell spreading in REF52 cells	[31]
	SH3, Proline-rich	Targets to FAs		[31,32]
	Arf GAP	Actin-based membrane ruffles	Facilitates Arf1 GTP/GDP cycles and actin remodeling necessary for *Salmonella* invasion	[26]
ASAP2	Arf GAP	F-actin structures at phagocytic cup	Regulates FcγR-mediated phagocytosis, potentially promotes by facilitating Arf6 GTP/GDP cycles	[12]
	BAR	Phagocytic cup association	Regulates FcγR-mediated phagocytosis under control of Selk	[33]
ASAP3	N.D.	Stress fibers	Facilitates stress fiber formation, migration and invasion of MDA-MB-231 cells	[34]
ACAP1	Arf GAP (partly)	CDRs	Inhibits CDR formation through GAP activity	[35]
	Arf GAP	Actin-based membrane ruffles	Facilitates Arf6 GTP/GDP cycles and actin remodeling necessary for *Salmonella* invasion	[26]
ACAP2	Arf GAP (partly)	CDRs	Inhibits CDR formation through GAP activity	[35]
	Arf GAP	Phagocytosis/phagocytic cups	Regulates FcyR- or zymosan-induced phagocytosis by facilitating Arf6 GTP/GDP cycles under control of Rab35 GTP/GDP cycles	[9,10]
	Ank	Rab35•GTP-dependent recruitment to phagocytic cups	Regulates FcγR-mediated phagocytosis under control of Rab35 GTP/GDP	[10]
	Arf GAP		Neurite outgrowth in PC12 cells	[36,37]
	Ank	Rab35•GTP-dependent recruitment to plasma membrane	Neurite outgrowth in PC12 cells	[37,38]
ACAP3	Arf GAP	Uni/bipolar morphology of migrating neurons	Promotes neurite outgrowth by facilitating Arf6 GTP/GDP cycles in hippocampal neurons	[39]
	Arf GAP	N.D.	Promotes neuron migration in developing cerebral cortex	[40]
ARAP1	PH3-PH4-Rho GAP-RA-PH5 (Arf GAP-independent)	Podosomes/sealing zones	Promotes dynamics and formation of podosome belt to aid bone resorption in osteoclasts	[41]
	Arf GAP	CDRs	Regulates CDR ring size and macropinocytosis in NIH3T3 fibroblasts	[42]
	PH3-PH4-Rho GAP-RA-PH5	CDR-targeting but not effect on CDRs		[42]
	Rho GAP, RA (mediates Rap•GTP binding)	Lamellipodia, focal complexes, stress fibers	Promotes the formation of leading edge structures in migrating NIH 3T3 fibroblasts	[43]
	Arf GAP	Filopodia	Promotes filopodia formation in NIH 3T3 and HEK293T cells by activating Cdc42 activation and controlling its distribution	[44]
	Rho GAP	Stress fibers	Moderates stress fibers in NIH3T3 cells	[44]
ARAP2	Arf GAP, Rho GAP (RhoA•GTP binding, not GAP activity)	Focal adhesions, stress fibers	Promotes focal adhesion growth and stress fiber formation in HeLa, MDA-MB-231, and U118 glioblastoma cells	[45,46,47]
	Arf GAP	Focal adhesions	Controls integrin β1 recycling in HeLa cells at APPL1 endosomes	[48]
	Arf GAP, Rho GAP (RhoA•GTP binding, not GAP activity)	F-actin structures around Listeria InB-coated beads	Promotes *Listeria* engulfment and F-actin enrichment around InB-coated beads	[49]
ARAP3	Rho GAP	Podosome-like adhesions	Mediates the response to a lack of traction forces in nontransformed fibroblasts on fluid surfaces	[50]
	Rho GAP	Filopodia, lamellipodia	Inhibits motility, invasion and adhesion of scirrhous gastric carcinoma cells	[51]
	N.D.	Lamellipodia, focal adhesions, stress fibers	Mediates the response of PAE cells to growth factor simulation	[52]
GIT1	N.D.	Podosomes	Promotes bone resorption activity in osteoclasts	[53]
	Arf GAP	Invadopodia	Facilitates the regulation of ECM degradation by Rac3 in MTLn3 cells	[54]
	SHD, PBS2	Growth cone	Regulates neurite extension and branching	[55]
	SHD	Lamellipodia	Promotes directional migration of endothelial cells towards VEGF	[56]
	N.D.	Podosomes	Mediates the response to VEGF and promotes ECM degradation and migration in endothelial cells	[57]
	SHD	Focal complexes/adhesions	Promotes focal complex disassembly and motility in fibroblasts and epithelial cells	[58]
	CC	N.D.	Enhances GRK6-mediated phagocytosis of apoptotic cells by inhibiting Rac1	[59]
GIT2	N.D.	Lamellipodia, focal adhesions	Inhibits lamellipodia formation, stabilizes focal adhesions and attenuates invasion of mammary epithelial cells	[60,61]
	N.D.	Filopodia	Induces filopodia in growth cones, promotes neurite branching in hippocampal neurons	[62]
	N.D.	Podosomes/sealing zones	Promotes podosome formation	[63]
AGAP1	Arf GAP (partly)	CDRs, stress fibers	Inhibits formation of CDRs and stress fibers	[64]
AGAP2	GLD (partly), (Arf GAP-independent)	Focal adhesions	Disassembly of FAs in HEK293, U87 and PC12 cells; promotes neurite outgrowth in PC12 cells	[65,66]

## Abbreviations

ALPS	Arf GAP1 lipid-packing sensor
Arf	ADP-ribosylation factor
Arf GAP	Arf GTPase-activating protein
Arls	Arf-like proeins
ARNO	ARF nucleotide-binding site opener
BAR	Bin/Amphiphysin/Rvs
CA	Constitutively active
CALM	CALM binding domain
CB	Clathrin-box
CC	Coiled-coil
CDR	Circular dorsal ruffles
Crk	Chicken tumor virus 10 regulator of kinase
DOCK	Dedicator of cytokinesis
DN	Dominant negative
ECM	Extracellular matrix
EGF	Epidermal growth factor
EFA6	Exchange factor for Arf6
ELMO	Engulfment and cell motility protein
ERK	Extracellular signal-regulated kinase
FA	Focal adhesion
F-actin	Filamentous actin/actin filaments
FAK	Focal adhesion kinase
GDI	Guanine nucleotide dissociation inhibitor
GEF	Guanine nucleotide exchange factor
GIT	G protein-coupled receptor kinase interactor
GLD	GTP-binding protein-like domain
GRK	G protein-coupled receptor kinase
Mtb	*Mycobacterium tuberculosis*
HGF	Hepatocyte growth factor
NGF	Nerve growth factor
NM2	Nonmuscle myosin 2
NM2A	Nonmuscle myosin 2A
N-WASP	neural Wiskott-Aldrich syndrome protein
PAK	p21-activated kinase
PBS	Paxillin binding site
PDGF	Platelet-derived growth factor
PH	pleckstrin homology domain
PIP	Phosphoinositides
PIX	PAK-interacting exchange factor
PI3K	Phosphoinositide 3-kinase
PI4P5K	Phosphatidylinositol 4-phosphate 5 kinase
PI(4,5)P2	Phosphatidylinositol 4,5-bisphosphate
PI(3,4,5)P3	Phosphatidylinositol 3,4,5-triphosphate
PLD	Phospholipase D
PMA	Phorbol 12-myristate 13-acetate
RA	Ras association motif
ROCK	Rho-associated kinase
SAM	Sterile α-motif
SARs	Secretion-associated and Ras-related proteins
SelK	Selenoprotein K
SHD	Spa-homology domain
SH3	Src homology 3 domain
VEGF	Vascular endothelial growth factor
WAVE	WASP family veroprolin homologous protein
WRC	WAVE regulator complex
